# Reproducible Reporting of the Collection and Evaluation of Annotations for Artificial Intelligence Models

**DOI:** 10.1016/j.modpat.2024.100439

**Published:** 2024-01-28

**Authors:** Katherine Elfer, Emma Gardecki, Victor Garcia, Amy Ly, Evangelos Hytopoulos, Si Wen, Matthew G. Hanna, Dieter J.E. Peeters, Joel Saltz, Anna Ehinger, Sarah N. Dudgeon, Xiaoxian Li, Kim R.M. Blenman, Weijie Chen, Ursula Green, Ryan Birmingham, Tony Pan, Jochen K. Lennerz, Roberto Salgado, Brandon D. Gallas

**Affiliations:** a United States Food and Drug Administration, Center for Devices and Radiological Health, Office of Science and Engineering Laboratories, Division of Imaging Diagnostics and Software Reliability, Silver Spring, Maryland; b National Institutes of Health, National Cancer Institute, Division of Cancer Prevention, Cancer Prevention Fellowship Program, Bethesda, Maryland; c Department of Pathology, Massachusetts General Hospital, Boston, Massachusetts; d System Development, iRhythm Technologies Inc, San Francisco, California; e Department of Pathology and Laboratory Medicine, Memorial Sloan Kettering Cancer Center, New York, New York; f Department of Pathology, University Hospital Antwerp/University of Antwerp, Antwerp, Belgium; g Department of Pathology, Sint-Maarten Hospital, Mechelen, Belgium; h Department of Biomedical Informatics, Stony Brook University, Stony Brook, New York; i Department of Clinical Genetics, Pathology and Molecular Diagnostics, Laboratory Medicine, Lund University, Lund, Sweden; j Department of Laboratory Medicine, Yale School of Medicine, New Haven, Connecticut; k Department of Pathology and Laboratory Medicine, Emory University School of Medicine, Atlanta, Georgia; l Department of Internal Medicine, Section of Medical Oncology, Yale School of Medicine and Yale Cancer Center, Yale University, New Haven, Connecticut; m Department of Computer Science, School of Engineering and Applied Science, Yale University, New Haven, Connecticut; n Department of Biomedical Informatics, Emory University School of Medicine, Atlanta, Georgia; o Department of Pathology, Center for Integrated Diagnostics, Massachusetts General Hospital, Harvard Medical School, Boston, Massachusetts; p Division of Research, Peter Mac Callum Cancer Centre, Melbourne, Australia; q Department of Pathology, GZA-ZNA Hospitals, Antwerp, Belgium

**Keywords:** Annotation Study, Artificial Intelligence Validation, Data set, digital pathology, Reference Standard, Reproducible Research

## Abstract

This work puts forth and demonstrates the utility of a reporting framework for collecting and evaluating annotations of medical images used for training and testing artificial intelligence (AI) models in assisting detection and diagnosis. AI has unique reporting requirements, as shown by the AI extensions to the Consolidated Standards of Reporting Trials (CONSORT) and Standard Protocol Items: Recommendations for Interventional Trials (SPIRIT) checklists and the proposed AI extensions to the Standards for Reporting Diagnostic Accuracy (STARD) and Transparent Reporting of a Multivariable Prediction model for Individual Prognosis or Diagnosis (TRIPOD) checklists. AI for detection and/or diagnostic image analysis requires complete, reproducible, and transparent reporting of the annotations and metadata used in training and testing data sets. In an earlier work by other researchers, an annotation workflow and quality checklist for computational pathology annotations were proposed. In this manuscript, we operationalize this workflow into an evaluable quality checklist that applies to any reader-interpreted medical images, and we demonstrate its use for an annotation effort in digital pathology. We refer to this quality framework as the Collection and Evaluation of Annotations for Reproducible Reporting of Artificial Intelligence (CLEARR-AI).

## Introduction

Reader-provided annotations of medical images are often used to support the evaluation of artificial intelligence (AI) and machine learning (ML) models. Wahab et al^1^ recently developed a systematic workflow for the construction, documentation, and evaluation of an annotation data set for digital pathology. We operationalized this workflow by creating a study development and reporting framework (Appendix A: the Collection and Evaluation of Annotations for Reproducible Reporting of Artificial Intelligence [CLEARR-AI] Reference Table) and corresponding checklist (Appendix B: CLEARR-AI Checklist) that can be used to aid data set creators in clearly reporting their work and by reviewers to evaluate the quality of a reported data set. In this article, we demonstrate the utility of this framework by applying it to our work for the high-throughput truthing (HTT) project,^2–5^ a multistakeholder, multidisciplinary project led by scientists at the US Food and Drug Administration’s Center for Devices and Radiological Health’s Office of Science and Engineering Laboratories.

The last 2 decades demonstrated explosive growth in the tools available to develop, use, and evaluate AI models for medical image analysis.^6,7^ Similarly, challenge competitions for medical image analysis helped drive momentum to develop and make available data sets and models for public access and understanding. Venues, such as the Medical Image Computing and Computer Assisted Intervention special interest groups,^8^ Grand Challenge,^9^ and Kaggle^10^ aggregate medical imaging data and analysis challenges across different diseases, imaging modalities, and image analysis tasks. The performance of the models generated from these challenges is measured against a reference standard – the true condition of the disease state. The reference standard, often referred to as “ground truth,” indicates the presence or absence of a diseased or abnormal condition and may include the location or extent of the condition.^11^ Often, the reference standard is generated from humans (eg, pathologists or radiologists). Here, “annotation” refers to any marks, segmentations, measurements, or labels made by a human reader of an image or image construct (images created to expand a data set or otherwise artificially generated^12^). Consistent and accurate annotation data sets are a crucial components to measure performance. To ensure reproducible results, the components of the annotation study, including metadata and image acquisition parameters, and the annotation methods should be reported clearly and consistently.

Several factors affect the reproducibility of AI models, including the model’s architecture, hyperparameters, loss function, weights initialization, training method, data preprocessing, AI output postprocessing, and training, tuning, and testing (validation) data sets. Transparent and quality reporting of AI/ML models as a unique component of studies is a topic of ongoing discussion in the Standards for Reporting Diagnostic Accuracy (STARD) and Transparent Reporting of a Multivariable Prediction model for Individual Prognosis Or Diagnosis (TRIPOD) communities.^13–16^ The proposed AI extensions to STARD and TRIPOD have yet to be made available, and it is unclear how much detail these extensions will recommend for reporting the creation of the components of human-annotated reference standards. AI extensions to the Consolidated Standards of Reporting Trials and Standard Protocol Items: Recommendations for Interventional Trials checklists are already adopted^17,18^ and do not address human observers. Similarly, Maier-Hien et al^19,20^ described the current transparency gaps in the reporting of AI/ML challenge competitions in the medical imaging communities, including the need for complete and reproducible reporting of data sets used in model development.^21,22^ Recently, Homeyer et al^23^ provided a broad review of issues related to compiling test data sets, from specifying the target population to data collection, annotation, and curation, and also bias detection, sample size, and regulatory requirements. AI diagnostic and detection models rely on training, tuning, and testing data sets for the development and evaluation of model performance. All types of data sets require accurate, transparent, and reproducible reporting of data set creation, necessitating the development of a reporting framework and the theme for this work.

A significant impediment to constructing annotated data sets is the number of annotations that must be collected from expert readers. Imaging data sets required for model development can be on the scale of thousands of images or more. The scale of this work is one reason why many researchers utilize crowdsourcing methods to recruit annotators from outside their immediate network.^24^ As researchers of these types of studies have demonstrated, using crowdsourced annotators recruited from the public requires additional care to ensure high-quality annotated data sets.^12,24,25^ Accurate reporting includes information about the annotators, such as recruitment methods and expertise about the specified annotation task. This information can be overlooked, although it is a critical component of transparency in data set reporting.

Having previously reported the HTT project’s approach to collecting crowdsourced pathologist annotations,^2^ we applied Wahab et al’s^1^ digital pathology workflow to our data collection methods and expanded its applicability to all forms of medical imaging. Wahab et al’s^1^ process describes an 11-step workflow: 10 steps of data set creation and a quality review step. We identified that this methodology could be utilized to develop a reporting framework generalizable to the entire medical imaging community. We refined Wahab et al’s^1^ process and gave it a name, Collection and Evaluation of Annotations for Reproducible Reporting of Artificial Intelligence models (CLEARR-AI). The goal of this manuscript was to demonstrate the usability of the CLEARR-AI framework with an existing project to improve transparency in reporting data sets for model development and evaluation by developers and hosts of image annotation repositories.

## Methods

### Description of the Collection and Evaluation of Annotations for Reproducible Reporting of Artificial Intelligence Framework

**Figure 1.**
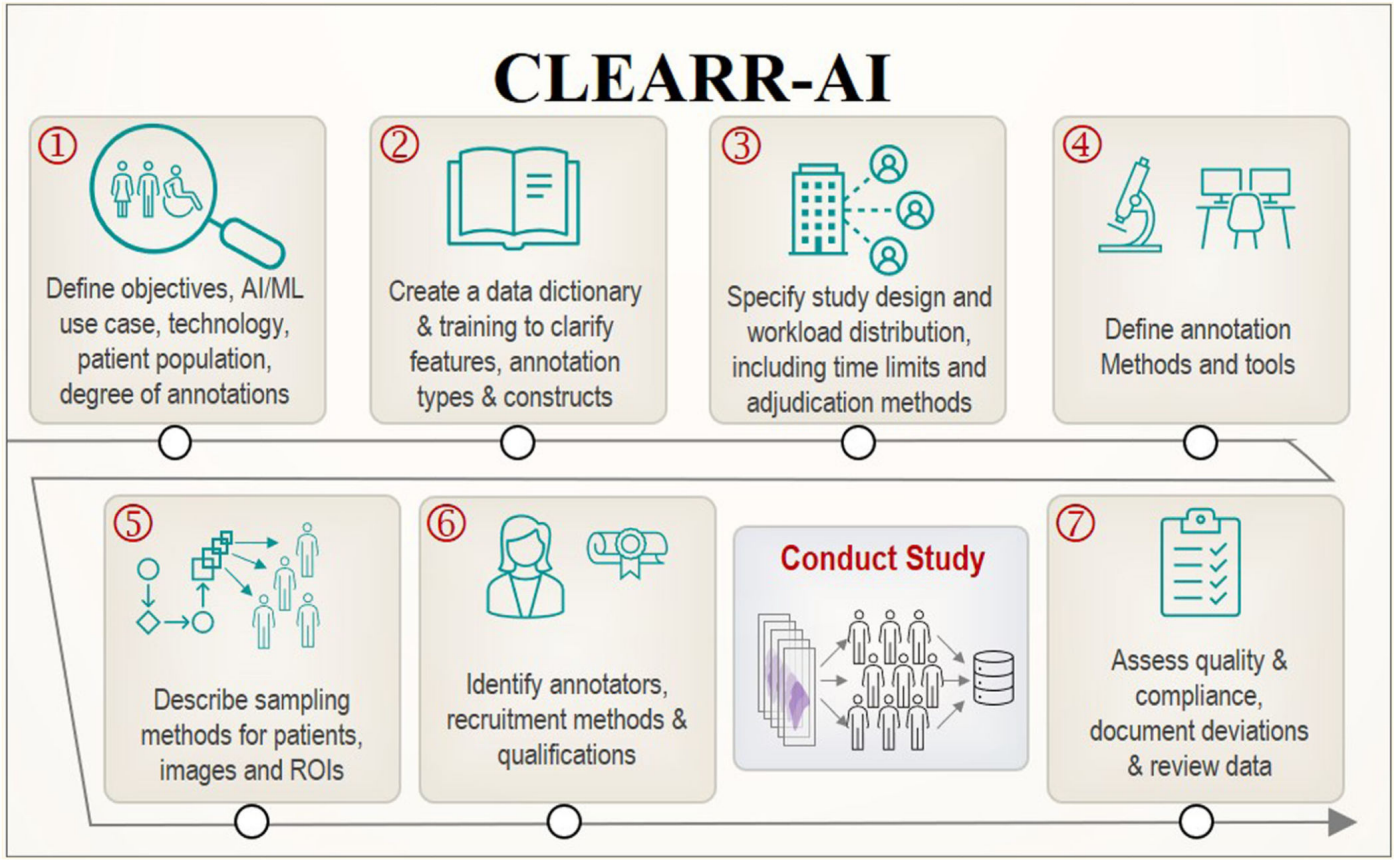
The Collection and Evaluation of Annotations for Reproducible Reporting of Artificial Intelligence (CLEARR-AI) framework.

The goal of the CLEARR-AI framework is to support many of the best-practice recommendations of Wahab et al^1^ while providing a consolidated reporting list that investigators can use to communicate the construction of medical image annotation data sets accurately and transparently. This template can be used in conjunction with other reporting guidelines such as STARD or Standard Protocol Items: Recommendations for Interventional Trials-AI. CLEARR-AI consolidates the original 11-step workflow into 7 essential components, summarized in Figure 1. Here, we describe each component of CLEARR-AI with an explanation of each step, including examples. Appendix A: CLEARR-AI Reference Table lists each of these 7 elements, provides a brief explanation of what each component entails, and connects each component to the original step in Wahab et al.^1^

### Objectives = CLEARR-AI Item 1

Define the project objectives and use case of the annotation data set. Consideration should be given to the intended use of models generated from the data set and any interactions of existing models with the data set. For example, data set objectives may state the intended use is for validation of detection and/or diagnostic models for well-defined clinical tasks. The objectives should specify the degree of annotations (regions of interest [ROIs] vs one region of an image (hot spot) vs entire image) needed for different units of analysis (pixel-level vs region-level vs image/patient level). As public sharing and reuse of data sets become increasingly common, we acknowledge that the data set may serve other use cases not originally identified by the study coordinators.

This step may best be accomplished by first describing a model that could use the data set, including its envisioned inputs and outputs. The description can shape annotation methods and data constructs and should be rooted in how the model will be used in current clinical environments to make patient management decisions. This description should include the definition of the data set’s intended clinical population including inclusion/exclusion criteria and patient-level demographics. The image acquisition systems (device manufacturers and models) and acquisition protocol (patient and specimen preparation, acquisition resolution, along with reconstruction and image processing methods) should also be included.

### Data Dictionary = CLEARR-AI Item 2

A data dictionary that includes definitions of metadata and annotation features is a necessary component for standard and reproducible annotations. The data dictionary may include a list of demographic and clinical information (age, gender, race, ethnicity, disease type, treatment, etc), the method used to identify the metadata (eg, patient-reported demographics; manual chart review; or specific laboratory tests; and/or medical codes applied to identify disease type, severity, and other biomarkers), and the expected format of each metadata field (eg, continuous data points and discrete list of possibilities).

Elements of the data dictionary will serve as a reference guide for annotators and identify the constructs that will be used for annotation. Annotations can be nominal, ordinal, quantitative, or a mixture thereof. The annotation constructs can either be restrictive (only annotate within a bounding box) or indicative (draw arrows, polygons around features, or boundary lines). The annotation construct may also vary based on the degree of annotation. A nominal label of a tissue section may be appropriate to describe a whole slide image (WSI), whereas it would be extremely time consuming to label every single cell within the same image.

The data dictionary may also include training materials and examples of annotations and their diagnostic/prognostic value, common annotation challenges and pitfalls, and reference items that will aid in uniform data collection. Written annotation instructions, checklists, controlled interface workflows, and training are valuable to collecting clean and consistent data. The data dictionary includes all training components available to annotators, both required and optional. When possible, these data dictionary can be created through consensus agreement with developers, expert clinicians, and representative annotators.

### Study Design = CLEARR-AI Item 3

The levels, constructs, and degree of annotation are all factors that will influence the annotation study design and workload for each annotator. Additional factors are the expected number of annotators (total, per case, per region, and feature), their experience level, their familiarity with the task, whether an annotator is permitted to edit a previous annotation, and whether any methods were employed to randomize the order that cases are presented to annotators. Adjudication of the experts’ annotations should also be described. In some cases, it may be efficient to employ junior staff or nonspecialists to make draft annotations that are subsequently checked, modified, and/or confirmed by a specialist or expert.

It is preferred for project creators to define the workload distribution before the start of the study. This planned distribution will ensure uniform and consistent data collection with an equitable allotment of annotations among images and annotators and reduce the risk that the annotator might not be able to finish the task. Furthermore, a thorough understanding of all workload factors may allow study designers to determine if annotators are encouraged to complete a task without time restrictions or whether annotators are expected to complete a task in a single sitting, which would be critical for a time-based comparator study.

### Annotation Methods = CLEARR-AI Item 4

Medical imaging data sets are constructed with different types of annotation methods. Annotators can make annotations by hand on a case report form, in a spreadsheet, with software tailored to record annotations, or a combination of all. Annotations are sometimes based on viewing digital images, such as x-ray imaging, magnetic resonance imaging, or reading medical reports. Some annotations might require in-person assessment by the annotator (slides on a microscope) and might also require the patient to be present (colonoscopy, ultrasound). Annotations may be made with the assistance of an AI/ML model external to the annotation study. It is important to describe annotation methods in detail and discuss how they align with the objective of the annotation study, field-specific reference standards, and existing regulatory and/or clinical frameworks.

### Image Curation = CLEARR-AI Item 5

After the data sources are identified, it is appropriate to describe the sampling strategies. For example, a data set might include all cases that satisfy the inclusion/exclusion criteria at one site in a specific time frame. Similarly, it may be appropriate to use sampling strategies that permit equitable representation of cases across all target populations and subgroups. Sampling methods may help balance or enrich the representation of different subpopulations for a more statistically efficient data set. If the study uses ROIs within images, the method of choosing them should also be described. If annotators are presented with a subset of the study’s entire image repository, how each subset was constructed should be described. When possible, and depending on a project’s objective, image data sets should contain a range of patient subgroups, image acquisition parameters, target features, and variability among the features. In contrast to Wahab et al’s^1^ recommendation that only the highest quality images be included in an annotation data set, it may be appropriate to include an assortment of images ranging from high quality to low quality to represent the images that will be encountered in the field. Actual medical images can be complicated by patient factors, physical and digitization artifacts, and/or inter- and intravariability in preparation sites. Incorporating a range of these types of images can be an important component of model validation. The definition of high- and low-quality images and how they should be distributed in the data set are dictated by the modality, target population, use case, and other components of the study’s objectives.

### Annotators = CLEARR-AI Item 6

Useful information to report about the annotators includes the annotator recruitment methods (including relationship to the developer), applicable annotator qualifications and requirements (such as mandatory certifications or training), and justification for inclusion and exclusion criteria for annotators. If an expert adjudicator is utilized, what qualifications differentiate an expert from other annotators should be specified. For example, creators of a pathology data set designed for use in a diagnostic model should consider whether annotators should be restricted to board-certified pathologists, or if pathology trainees (eg, residents and fellows), researchers, and/or pathology extenders (eg, histotechnologists) who are skilled in creating annotations, are also eligible. This documentation may include board certification information, years of experience, whether annotators have taken any special training and other similar information.

### Quality Review = CLEARR-AI Item 7

Throughout and after the completion of the study, study coordinators should review the preceding steps and resulting data set for areas of improvement. Study coordinators should identify key points during the study to conduct an interim analysis of the performance of the above components. The analysis includes identifying modifications that may have occurred during the study to the time point, why the modification was made, and how data analysis will account for the modification. Upon completion of the study, this review includes an assessment of the completeness of the study, such as whether all images were annotated according to the study protocol. For small studies that are part of a larger project, such as a pilot study followed by a pivotal study, then study coordinators should also identify what additional steps will be needed to resolve any issues before proceeding to the next phase of the study.

## Results

### Application of Collection and Evaluation of Annotations for Reproducible Reporting of Artificial Intelligence to the High-Throughput Truthing Project

We demonstrate the CLEARR-AI reporting framework by applying it to the pilot study of the HTT project. Besides documenting our pilot study, we identify shortcomings in our pilot study annotation methods and the necessary improvements for our pivotal study. We comment on these shortcomings throughout and discuss the changes made for the HTT pivotal study (launched June 2023) in the Quality Review section.

### Objectives = CLEARR-AI Item 1

The purpose of the HTT project is to create a validation data set of pathologist-annotated ROIs in glass slides and WSIs of triple-negative breast cancer (TNBC) biopsies^2–5^ stained with hematoxylin and eosin (H and E). The primary annotation collected on each ROI is the density of stromal tumor-infiltrating lymphocytes (sTILs),^26^ which is a quantitative biomarker that is prognostic for survival in TNBC^27–29^ and other tumor types.^30–32^ The objectives of the pilot study (February 2020 to May 2021) were to test the study design, annotation methods, and analysis methods that would assist us in sizing, executing, and analyzing the pivotal study.^2^ Of these objectives, it was most important to understand pathologist variability, so we could account for it with the analysis methods and sizing for the pivotal study.

We envision that the computational model that will use this validation data set would be used to provide information for making management decisions, including therapy. The model’s input will be ROIs selected for analysis by a pathologist, and the primary output will be the density of sTILs in the ROI. The data set will be available to developers for testing models with sequestration controls in place to track and limit the use of the data set to avoid biases associated with training to the test data. A secondary objective of the pilot study was to explore annotations made with glass slides on an optical microscope vs those made with a WSI using a digital viewing system. These systems are discussed in the Annotation Methods section.

At the time of the pilot study, the inclusion and exclusion criteria for the clinical population and the data set were not explicitly specified, including the focus on TNBC cases. As the primary objective of the pilot study was to understand pathologist variability, and we were at the beginning of our collaborative relationship, we used a convenient set of slides and images for the pilot study. The set of slides obtained were breast cancer biopsies with no additional metadata; more details are discussed in the Image Curation section. Please see the Quality Review section for information on the inclusion and exclusion criteria and the patient metadata collected for our pivotal study.

### Data Dictionary = CLEARR-AI Item 2

The initial data dictionary of the pilot study of the HTT project was developed by the project members and consisted of a manuscript, a recorded tutorial, and an informed consent document. The manuscript is from the International Immuno-Oncology Biomarker Working Group^33^ and provides recommendations for the evaluation of sTILs in breast cancer. The recorded tutorial is about the sTILs evaluation process discussed in the “sTILs evaluation” manuscript,^34^ and Figure 4 from the “sTILs evaluation” manuscript was also made available to assist annotators. We refer to the figure as an sTIL calibration reference guide; it shows example ROIs and related sTILs density values to help pathologists calibrate their scores. These materials were available to participants on a wiki page that was titled, “HTT Data Collection Training: Pilot Study.” The training materials covered the annotation target feature (eg, sTILs) and how to use the annotation technology. During the quality review of the pilot study, we decided that the training methods needed improvement and tracking. These changes are discussed later in the Quality Review section.

Annotations are restricted to ROIs. Each annotation for an ROI has 3 components defined in the training. The first annotation applied 1 of 4 nominal labels of ROI type based on whether the ROI is evaluable for sTIL evaluation (tumor with intratumoral stroma or invasive margin tissue types) or not evaluable for sTILs (tumor with no intervening stroma or other region). The second and third annotations were only carried out if the ROI was labeled as evaluable for sTIL annotation. The second annotation was a quantitative input of the percentage (%) of tumor-associated stroma in the ROI. This annotation was added to the pilot study midway to help the pathologist understand the denominator of the third annotation. The third annotation was a quantitative input of the estimated area of sTILs as a percentage (%) of the area of tumor-associated stroma.^2,3^

Annotators for the HTT project provided ROI-level annotations. To demarcate the area for evaluation, a 500 × 500 μm bounding box was superimposed on the predetermined annotation area either through a physical reticle in the microscope objective or a virtual box on top of the WSI. The annotation data were aggregated from the different platforms, cleaned, and saved in human-readable form in a spreadsheet (comma-separated values) and in analysis-ready form as an R data frame object named “pilotHTT.” These data are part of an R data package that also includes analysis scripts that produce figures for presentations. The R data package is publicly available under version control, including data documentation.^35^

### Study Design = CLEARR-AI Item 3

Although the annotation task itself only takes approximately 30 seconds per ROI,^18^ evaluating a large number of ROIs to create a validation data set is a time-consuming request of volunteer pathologists. Therefore, to reduce the burden on our volunteer pathologists and to manage the workflow, the total number of pilot study ROIs (n = 640) were divided into 8 batches of 8 WSIs with 10 ROIs per WSI (80 ROIs per batch). The pilot study goal was to collect annotations from at least 5 pathologists per ROI. We asked (but did not enforce) annotators to use a random number generator to select batches, complete selected batches, and complete as many batches as they could.^3,4^ We took more control of the study design and workload distribution in the pivotal study (see the Quality Review section), because we found one limitation in our pilot study was that the distribution of pathologists per ROI was not balanced.^3^ There were no limitations on how long a pathologist had to do annotations. They could stop annotating in the middle of a batch or an image and return later. They were not permitted to change an annotation after it was saved.

### Annotation Methods = CLEARR-AI Item 4

The HTT Pilot Study used custom-built annotation platforms to control data collection. Pathologists provided annotations for the HTT project using 1 of 2 modalities: in-person using a glass slide on an optical microscope or remotely using a Web application for WSI viewing and annotation.^3^ The in-person data collection platform is called evaluation environment for digital and analog histopathology (eeDAP). eeDAP uses custom hardware–software to control the microscope field of view and visit prespecified ROIs.^36,37^ The 2 digital platforms were HTT-custom instances of PathPresenter^38,39^ and caMicroscope.^40^ The annotations were recorded by the microscope and digital platforms during the annotation study. The pathologists worked independently and there were no adjudication methods applied. Pathologists were free to choose which platform to use, but the eeDAP platform became unavailable during the COVID-19 pandemic.

### Image Curation = CLEARR-AI Item 5

The deidentified slides and images for the pilot study were prepared and provided by the Institut Jules Bordet (Brussels, Belgium). There was no additional quality review of the slides or images after the slides were received. No patient information (demographic or clinical) was provided. As such, there were no efforts to be equitable across patient subgroups. Please refer to the Quality Review section for significant changes made to data curation about patient subgroups for the pivotal study.

All 64 slides in the pilot study were from breast cancer biopsies of invasive ductal carcinoma stained with H and E. Slides were scanned on a Hamamatsu Nanozoomer 2.0-RS C10730 series at ×40 equivalent magnification (0.23 μm/pixel). A collaborating pathologist was instructed to select 10 ROIs from each slide with a wide variety of morphologic features with a distribution of tissues that did and did not contain the target feature (sTILs). The protocol for selecting ROIs can be found in the work of Dudgeon et al.^2^

### Annotators = CLEARR-AI Item 6

Volunteer pathologists were recruited using crowdsourcing methods by posting project advertisements on research boards, in pathologist listservs, and through collaborator word of mouth. Referencing our project objective, we recruited board-certified pathologists and pathology residents and fellows, and offered training, as described in the Data Dictionary. We implemented a registration survey for annotators to collect information on their title (practicing pathologist, a resident, or fellow), number of years of experience, and any board certifications (or equivalent for non-US pathologists). Some participants did not complete the registration survey, and the training was not monitored or verified. Although annotators were uniquely identified in the pilot study based on their login, not monitoring and tracking participant training and registration was a shortcoming in the pilot study that we fixed for the pivotal study (see the Quality Review section). There were 27 annotators in the pilot study (August 2021): 13 pathologists, 4 residents, and 10 “unknown.” As mentioned in the Study Design section, the number of pathologists per ROI was not controlled, and therefore, was not balanced.^3^ We discuss the new controls implemented in the pivotal study in the following section.

### Quality Review = CLEARR-AI Item 7

The HTT Pilot Study was the first phase of a 2-phase study, where the second phase is the pivotal study. We conducted a review of our pilot study after its conclusion in May 2021,^3^ we completed a subsequent deep dive expert panel review in the Fall of 2021,^4^ and we launched the pivotal study in June 2023. This review allowed us to improve our protocols, tools, and plans for our pivotal study. Here, we identify and review those changes.

We also received feedback from the FDA Medical Device Development Tool program.^41^ Probably the most impactful feedback we received was about the data set use case and clinical population. The feedback recommended that we focus on TNBC samples, because there could be morphologic differences with other subgroups that could impact computational pathology models. The reason this feedback was impactful is that a chart review was needed and the prevalence of TNBC is only 12%.^42^ The feedback also identified patient demographics and disease severity as important characteristics that should be considered. We refer to all these characteristics as patient metadata and note that the metadata listed below has not yet been reviewed by the FDA.

Based on the feedback, we established the inclusion and exclusion criteria and the metadata that we require for our pivotal study samples (CLEARR-AI Reference Table: Objectives). We include H and E-stained slides of core biopsies of TBNC obtained in the last 7 years. We exclude any tissue collected after administration of any antitumoral or neoadjuvant therapy (eg, chemotherapy and radiation therapy). The demographic and clinical characteristics of the patient population that we are collecting are given in Table.^43^ We also collect the scanner make, model, specimen collection site, slide preparation site, and slide scanning site. This contrasts with the pilot study slides for which we only knew that the cases were breast cancer.

We also updated the use case of the image acquisition systems to prioritize data collection using the reference standard technology, the microscope. The primary data collection method will be the eeDAP microscope system, and work is underway to validate that the annotations collected with the microscope can be accurately mapped to the ROIs in the digital images. The digital platforms will be considered secondary data collection methods, because we will be using WSI viewers that are not FDA-cleared medical devices. We will use the results of the pivotal study to demonstrate that annotations collected with digital images and platforms are equivalent to annotations collected on the microscope. We will use the FDA-cleared Aperio AT2 DX WSI scanner to digitize the slides (×40 apparent magnification; 0.75 numeric aperture, 0.2525 μm/pixel).

Analyses of the pilot study annotations found that reader variability was high^3,4^ prompting us to make substantial upgrades to our annotator training materials (CLEARR-AI Reference Table: Data Dictionary).^4^ The analysis included roundtable discussions of each ROI, the collection of annotations, the writing of a paper summarizing the discussions,^4^ and the creation of a free and web-accessible continuing medical education (CME) course to educate pathologists with the assessment of breast cancer sTILs.^44^

The CME course gives the clinical context of sTILs in breast cancer, describes the assessment method in a short video,^45^, requires a review of the manuscript with the recommendations for the evaluation of sTILs in breast cancer,^33^ and reviews pitfalls accompanied by example images (source material from Garcia et al^4^). Passing the CME course is a requirement of annotators in the pivotal study, which we can confirm with the CME course certificate. This addresses the limitation in the *Annotators* component of our pilot study that we did not monitor or enforce the completion of training in sTILs assessment.

**Figure 2.**
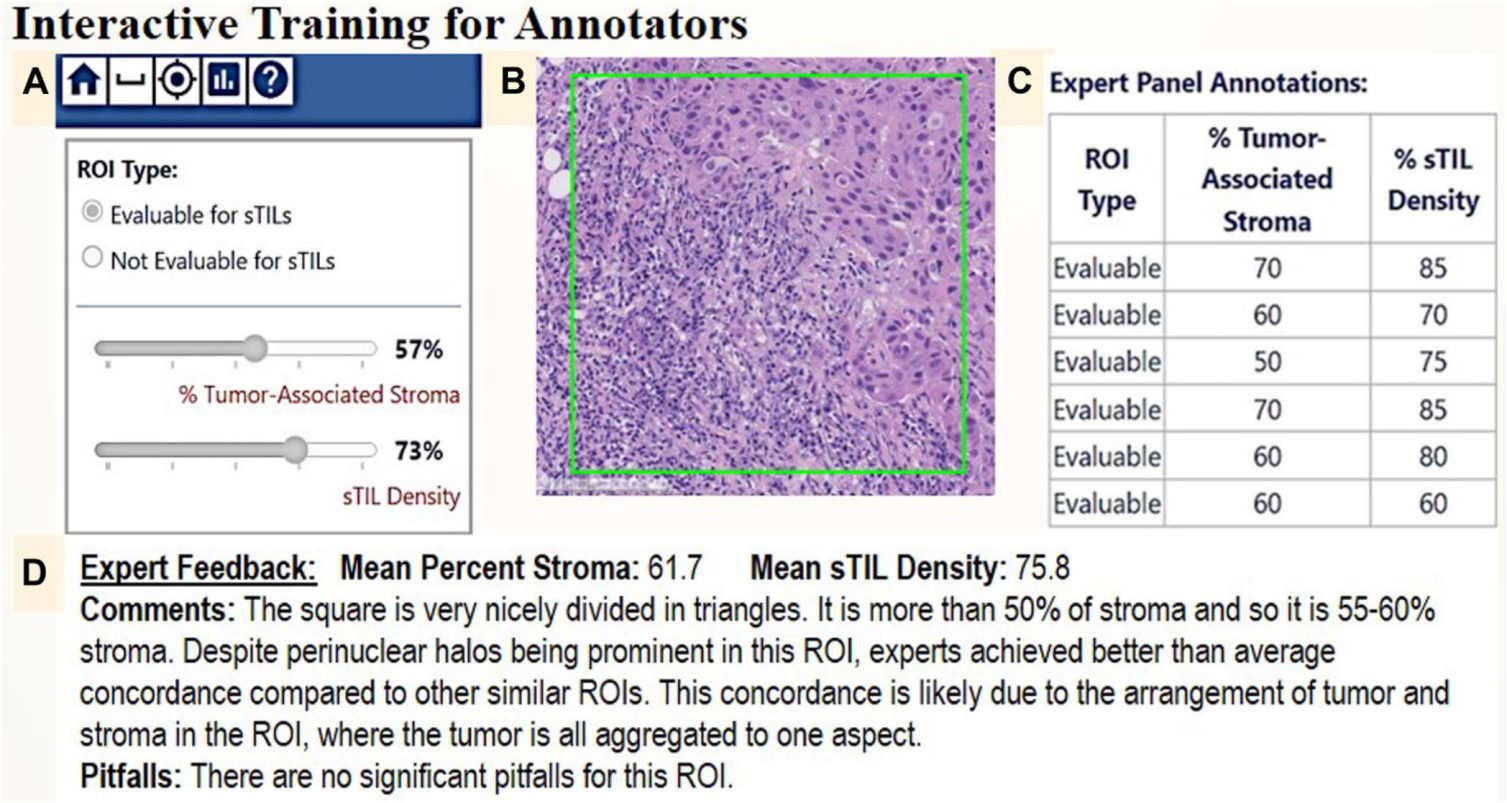
Example of interactive training module for sTILs Assessment: Test with Feedback. This module allows pathologists to make an annotation of a displayed ROI’s evaluability for sTILs assessment, percent tumor-associated stroma, and sTILs density. Upon saving the annotation, the pathologist can compare their score against that of 6 experts and review comments and pitfalls as described by the experts.^4^ ROI, region of interest; sTIL, stromal tumor-infiltrating lymphocyte.

We also created 2 interactive training modules, a Test with Feedback (Fig. 2) and a Proficiency Test. These tests are also derivatives of the deep dive by our expert panel. In the Test with Feedback (CLEARR-AI Reference Table: Data Dictionary), the participants use an augmented version of the existing digital annotation workflow to view and annotate ROIs. For each ROI, when the user clicks to save their annotations, they are provided the annotations from the expert panel along with commentary and a list of pitfalls curated by the expert panel. The performance on the Proficiency Test will be assessed, and if the participant passes, they will be qualified as an expert annotator for the pivotal study (CLEARR-AI Reference Table: Annotators). Pathologists also receive summary performance reports so that they can understand the performance assessment and how their scores compare with the expert panel.^46^

Postpilot study review also demonstrated the need for improvement in our data collection platforms to control workload distribution and improve our registration methods to obtain annotator information pertinent to the study.^18^ We updated the platforms to allow project administrators to control which batches a user can access to ensure equal annotation across batches (CLEARR-AI Reference Table: Study Design and Workload Distribution). This update also allows administrators to confirm that the pathologist has passed the Proficiency Test and that the registration information is complete (CLEARR-AI Reference Table: Annotators).

For our pivotal study, we designed an *Image Curation* process based on the metadata we are collecting. The motivation behind the new process was to create a data set that represents a spectrum of sTIL densities and includes relevant clinical features. The process involves prioritizing ROIs with medium and high-sTIL densities, because these ROIs were seen to be underrepresented in the pilot study. We make this possible by collecting “first-pass” primary annotation information (ROI type, percent stroma, and density of sTILs) during ROI selection. We also prioritize underrepresented demographic subpopulations and highlight common pitfalls along with the density of sTILs using a hierarchical rank-sort method. The results demonstrate improved distributions and successful inclusion of medium and high-sTIL density ROIs in the selected data set. The specific protocol will be shared elsewhere.

**Figure 3.**
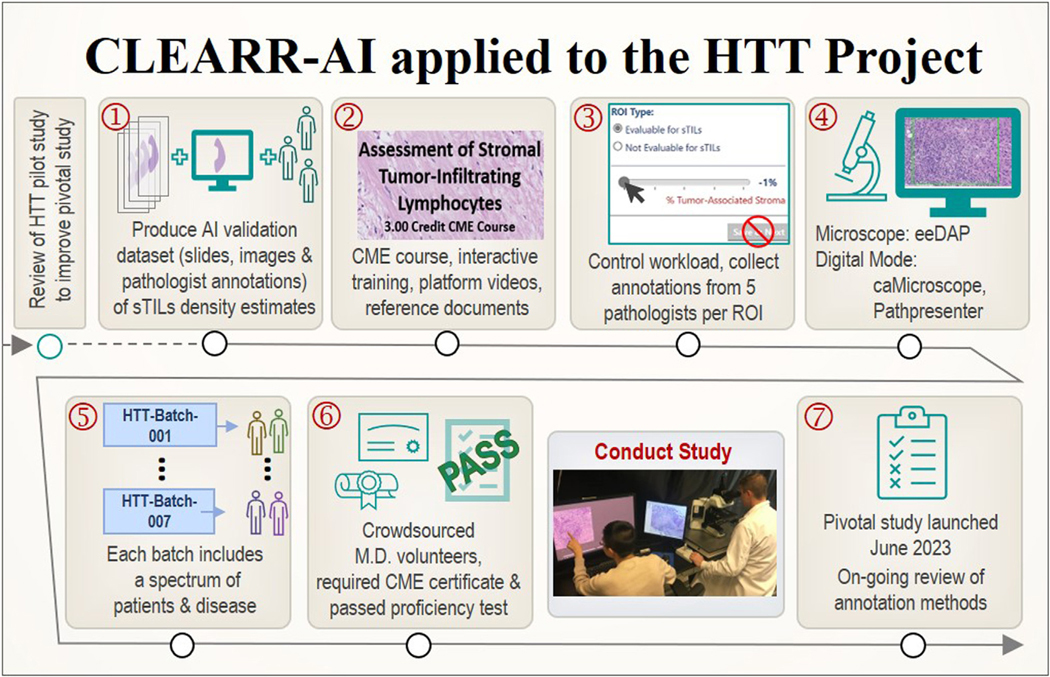
HTT pivotal study within the CLEARR-AI framework. CLEARR-AI, The Collection and Evaluation of Annotations for Reproducible Reporting of Artificial Intelligence; HTT, high-throughput thruthing.

We launched the pivotal study in June 2023 with the improvements, as described in this section. In Figure 3, we demonstrate a visual report of the HTT pivotal study using the CLEARR-AI framework.

## Discussion

### Collection and Evaluation of Annotations for Reproducible Reporting of Artificial Intelligence Framework

We consolidated several steps of the Wahab et al^1^ workflow into a list applicable to a wide range of annotation projects. We also redefined some of the steps to produce medical image annotation data sets that reflect real-world data, such as the inclusion of both high- and low-quality images, and documentation of annotator recruitment and experience information. We refer to this reporting framework as CLEARR-AI. CLEARR-AI is to be used in conjunction with planning and executing studies and preparing communications (manuscripts and grant proposals) to ensure clarity about data set creation and to facilitate the reproducibility of results.

### Application of Collection and Evaluation of Annotations for Reproducible Reporting of Artificial Intelligence to High-Throughput Truthing

To demonstrate the utility of CLEARR-AI, we reported on the HTT project’s pilot annotation study using the reporting framework and verified inclusion of all components with the associated checklist (Appendix B: CLEARR-AI Checklist). By completing all 7 components of the CLEARR-AI table, we reported the key elements of the pilot study of the HTT project to facilitate understanding by others. Furthermore, in step 7 of the framework, Quality Review, we described the changes from the pilot study that were implemented for the pivotal study. Based on this work, creators of human-annotated data sets can choose to utilize the tabular template of Appendix A: CLEARR-AI Reference Table to aid in preparing data sets (which refers to the work of Wahab et al^1^ that includes additional perspectives and examples) or follow the CLEARR-AI Checklist, (Appendix B), to maintain transparency, consistency, and reproducibility when reporting their work.

### Limitations of this Work

A limitation of this work is that we demonstrated comprehensive reporting of an annotation study using only 1 annotation study (HTT) with only 1 medical imaging domain (digital pathology). The HTT project is a collaboration of investigators highly familiar with the data collection methods and reporting necessary to create a reproducible and repeatable data set. The examples and perspectives in this work combined with those in Wahab et al^1^ can provide clarity to the reporting of any annotation study and may be used by future data set developers to improve study structure and reporting during their development process.

Another limitation of this work is that the framework is not the result of a rigorous consensus process. Although authors include collaborators from government, academic, and industry sectors familiar with the need for transparency in reporting AI/ML data sets, they do not represent the full breadth of the AI/ML community. Other reporting standards, such as STARD and TRIPOD, rely on input from all stakeholders in the community and a rigorous process with correspondingly large duration for comments and feedback in the construction of checklists. In the future, adoption and feedback of reporting standards by other data set developers and users in the medical imaging community will likely lead to improvements in reporting consistency and clarity.

### Relevance to the Artificial Intelligence Community

The recent advancements in AI/ML modeling and exponential growth in digital health technology are expected to lead to a surge in submissions of AI models as software as medical devices to regulatory agencies. Clarity in data set reporting is important even if the data set is not part of the medical device or the model developer did not create the data set(s) used in the submission. Repositories of medical image challenges, such as Grand Challenges and Kaggle may establish reporting standards, similar to this framework, for some or all data sets used for their challenges. Challenge platforms using data sets characterized with complete and detailed information similar to the annotation-reporting framework described in this article may be leveraged to produce evidence in support of regulatory submissions for medical AI. As the importance of transparency in the reporting of data sets becomes more apparent, we expect to see a corresponding standardization of reported data set components. The CLEARR-AI framework is relevant to the creation of training, tuning, and testing data sets. Training data sets can be very large and annotations less rigorous than tuning and testing data sets, but transparent reporting of training data set construction is equally important as for other data sets.

We hope that those assembling annotated data sets find this work useful and improve upon the reporting template through application to their annotation studies. We put forth and demonstrated the utilization of a study design framework and reporting checklist for annotation studies in medical imaging. We refer to this template as CLEARR-AI. We utilized this framework by reporting on our HTT project, which is creating a pathologist-annotated data set for the evaluation of AI/ML models in digital pathology.

## Supplementary Material

supp

supp2

The online version contains supplementary material available at https://doi.org/10.1016/j.modpat.2024.100439

## Figures and Tables

**Table:** HTT pivotal study metadata

Feature	Description	Possible values

Age	Age of patient at the time of sample acquisition. If the patient is aged > 189 y, age reported as 90^a^	Continuous whole numbers
Gender	Patient’s gender as defined in medical records. There was no differentiation for gender	Female Male
Race	Patient’s race. More than one response allowed	American Indian or Alaska Native; Asian; Black or African American; Native Hawaiian or Other Pacific Islander; White
Ethnicity	Patient’s ethnicity	Hispanic or Latino; Not Hispanic or Latino
Breast cancer stage	Denotes breast cancer stage (reflection of tumor size, lymph node positivity, and presence of metastases) at the time of biopsy	0; I; II; III; IV

This Table lists the clinical and demographic characteristics of the patient population requested from each site providing H and E slides for the HTT pivotal study. H and E, hematoxylin and eosin; HTT, high-throughput thruthing.

a In accordance with the HIPPA’s Privacy Rule^43^ the age of all patients aged >89 years is reported as 90 years.

## Data Availability

The pilot study and expert annotations are available at this public repository: https://github.com/DIDSR/HTT
