# Supplementary material for: Reproducible Reporting of the Collection and Evaluation of Annotations for Artificial Intelligence Models
Source: Mod Pathol. Author manuscript; Available in PMC 2026 Feb 16. (PMC12908141; doi:10.1016/j.modpat.2024.100439)
Supplement: supp [file NIHMS2133978-supplement-supp.docx]

# **Appendix A: CLEARR-AI Reference Table**

This table provides a concise description of each component of CLEARR-AI. It also indicates whether the component was originally described in Wahab *et. al.* [1] workflow and improved upon here or newly introduced in this manuscript.

| Study Component | Explanation | Origin | |
| --- | --- | --- | --- |
| 1. Objectives | Project objectives, dataset use case (training, testing models), degree of annotation (patient, image, ROI, or feature) and patient population. | Wahab *et al.* 1. Objectives and 2. Diagnostic/ Prognostic Algorithm (modified) and 7. Degree of Annotation |  |
| 2. Data Dictionary | Training materials and reference documents(s) describing image features, anatomic/biological context, and details on annotations:   - Types of annotation: nominal, ordinal, quantitative, or a mixture thereof; - Constructs: arrows, outlines, slider bars, text boxes). | Wahab *et al.* 3. Annotation Data Dictionary (modified), 5. Annotation Levels, and 6. Annotation Constructs |  |
| 3. Study Design | Specify the study design   - Number of annotators, number of cases, number of annotators per case - Methods to randomize cases or viewing conditions - Time limits for annotation tasks - Methods to assist or combine annotations (adjudication) | Wahab *et al.* 9. Workload Distribution (modified) |  |
| 4. Annotation Methods | Determine how annotators will encounter the data (digital platforms, in-person viewing, or mixed-methods), and what tools will be used to access and view the images. | Wahab *et al.* 4. Selection of Annotation Software, (modified) |  |
| 5. Image Curation | Specify annotator, patient, and image sampling methods for the entire study and individual sub-groups of a study (e.g. e.g. case sampling methods including enrichment, and stratified sampling):   - Annotator: exhaustive in an allotted time-period, random subset, or stratified sampling of subgroups; - Patient: demographic and clinical sub-groups, target features, and variability of features case sampling methods including enrichment, stratified sampling, and ROI selection; - Image: quality and number of images in each subgroup. | Wahab *et al.* 11. Quality Review (modified) |  |
| 6. Annotators | Define annotators: number of total annotators, number of annotators per case, qualifications (training and requirements), and how they were recruited. | [ New ] |  |
| 7. Quality Review | During and after the annotation study, identify, review and discuss adherence to the above components of the template, report the collected data, and report any deviations.  Specify whether this was a single study or part of a larger study. | Wahab *et al.* 8. Phase of Annotation and 11. Quality Review (modified) |  |
