# Supplementary material for: Reproducible Reporting of the Collection and Evaluation of Annotations for Artificial Intelligence Models
Source: Mod Pathol. Author manuscript; Available in PMC 2026 Feb 16. (PMC12908141; doi:10.1016/j.modpat.2024.100439)
Supplement: supp2 [file NIHMS2133978-supplement-supp2.docx]

# **Appendix B: CLEARR-AI Checklist**

| Section & Topic | # | Item |
| --- | --- | --- |
|  |  |  |
| Title or Abstract | **1** | Identifies the creation of an annotated dataset using an image-based modality (Radiology, Pathology, multi-modality, etc.) |
| 1. Objectives | **2** | Identifies dataset use case (training, tuning, or testing) |
|  | **3** | Identifies model inputs |
|  | **4** | Identifies model outputs |
|  | **5** | Identifies intended population (i.e. inclusion criteria, exclusion criteria of the dataset, demographic and clinical metadata) |
|  | **6** | Identifies image acquisition systems (i.e. modality, manufactures and models) |
|  | **7** | Specifies input image acquisition protocol (e.g. patient and/or specimen preparation, image processing and/or reconstruction methods) |
|  | **8** | Defines degree of annotation (list of all components being annotated: entire image, region of interest, or specific feature) |
| 2. Data Dictionary | **9** | Identifies and defines features that need to be annotated |
|  | **10** | Identifies metadata (demographic; clinical) for each case and method of identification of each category (i.e. patient-reported, manual chart review, or specific laboratory tests). |
|  | **11** | Identifies the diagnostic/prognostic value of each annotation type |
|  | **12** | Provides examples of relevant features with illustrative images |
|  | **13** | Provides examples of common pitfalls with illustrative images |
|  | **14** | Identifies annotator training methods and reference documents |
|  | **15** | Defines types of annotations (i.e. nominal, ordinal, quantitative and/or a mixture) |
|  | **16** | Defines annotation constructs (e.g. indicative arrows, restrictive bounding boxes, or free-hand marks) |
|  | **17** | Defines the format of data structures in the study (e.g. DICOM, list, etc.) |
| 3. Study Design | **18** | Defines number of annotators, number of cases, annotators per case and expected workload of each annotator |
|  | **19** | Specifies whether annotation task is time-limited and whether annotators can edit prior annotations |
|  | **20** | Identifies methods that include an assistant to the annotator or that combine annotations from annotators (adjudication methods) |
| 4. Annotation Methods | **21** | Specifies annotation method (i.e. in-person, digital, or a combination) |
|  | **22** | Specifies annotation technology (e.g. manufacture, model, version) |
|  | **23** | Specifies any AI tools that may provide draft annotations |
| 5. Image Curation | **24** | Identifies patient and image sampling methods to select any subset of images for annotation (e.g. case sampling methods including enrichment, stratified sampling, and ROI selection) |
|  | **25** | Patient: demographic and clinical sub-groups, target features, and variability of features case sampling methods including enrichment, stratified sampling, and ROI selection |
|  | **26** | Image: quality and number of images in each subgroup |
|  | **27** | Specifies whether images were excluded from annotation for any reason |
| 6. Annotators | **28** | Identifies annotators |
|  | **29** | Identifies annotators requirements and qualifications (e.g. credentials, experience, training) |
|  | **30** | Identification of annotator recruitment methods |
| 7. Quality Review | **31** | **Identifies whether review was conducted throughout the study, at specific checkpoints, at the conclusion of the study, or a mixed method.** |
|  | **32** | Identifies mid-study modifications to the above components. |
|  | **33** | Discusses adherence of study design and reporting to the CLEARR-AI framework |
|  | **34** | Specifies the phase of the annotation study in relation to a larger project |
